# Modulation of Animal and Human Hematopoiesis by β-Glucans: A Review

**DOI:** 10.3390/molecules16097969

**Published:** 2011-09-15

**Authors:** Michal Hofer, Milan Pospíšil

**Affiliations:** Laboratory of Experimental Hematology, Institute of Biophysics, v.v.i., Academy of Sciences of the Czech Republic, Královopolská 135, CZ-61265 Brno, Czech Republic

**Keywords:** β-glucan, hematopoiesis, myelosuppression

## Abstract

β-Glucans are cell wall constituents of bacteria, yeast, fungi, and plants. They are not expressed in mammalian cells, but they are recognized by mammalian cells as pathogen-associated molecular patterns by pattern recognition receptors and thus act as biological response modifiers. This review summarizes data on the hematopoiesis-stimulating effects of β-glucans, as well as on their ability to enhance bone marrow recovery after an injury. β-Glucans have been shown to support murine hematopoiesis suppressed by ionizing radiation or cytotoxic anti-cancer therapy. They also enhance stem cell homing and engraftment. Basically, two forms of β-glucan preparations have been investigated, namely particulate and soluble ones. β-Glucans are generally well tolerated, the particulate forms showing a higher incidence of undesirable side effects. Taken together, the hematopoiesis-stimulating properties of β-glucans predetermine these biological response modifiers to ever increasing use in human medicinal practice.

## 1. Introduction

In 1941, Pilemer and Ecker [1] applied the name zymosan to a *Saccharomyces cerevisiae* cell wall fraction which produced hyperplasia and hyperfunction of the reticuloendothelial system when administered to experimental animals. β-Glucan, a polysaccharide consisting of chains of glucopyranose units joined by 1-β glucoside linkages, has been isolated from zymosan and shown to be responsible for most of its stimulatory effects on the reticuloendothelial system [2,3]. Nowadays, β-glucans are known as cell wall constituents of bacteria [e.g., 4], yeast [e.g., 5], fungi [e.g., 6,7], and plants [e.g., 7,8]. They are not expressed in mammalian cells, but β-glucans are recognized by mammalian cells as pathogen-associated molecular patterns by pattern recognition receptors and thus act as biological response modifiers [9].

In the 1980s β-glucans were shown to act as broad-spectrum enhancers of host defence mechanisms [10,11]. This fundamental statement has been based on experimental results showing the ability of β-glucans to positively influence the immunological status of mammals during infections of bacterial [e.g., 12,13], viral [e.g., 14], and fungal [e.g., 15,16] origin. The anti-infective potential of β-glucans was reviewed in 1996 by Williams *et al.* [17].

The immune and hematopoietic systems are closely interconnected [18,19], therefore the attention of researchers investigating glucan effects has been also focused on the consequences of administration of β-glucans on hematopoietic cells in their total hierarchical spectrum from pluripotential stem cells through progenitor and precursor cells to mature functional peripheral blood cells. This review summarizes the findings on the hematopoiesis-modulating action of β-glucans and underlines the possibilities of their practical utilization in corresponding therapeutic areas, which cover especially utilization in conditions of myelosupression induced by ionizing radiation or cytotoxic anti-tumor therapy. Since two principal β-glucan forms, a particulate and a water-soluble one, have been prepared and tested, a separate chapter of this review is devoted to the concordances and differences in their effects.

## 2. Effects of β-Glucans on Normal Hematopoiesis

Early studies on the effects of β-glucans on hematopoiesis in otherwise untreated experimental mice were performed in the late 1970s. These studies showed that particulate β-glucan possesses the ability to increase the number of granulocyte progenitor cells in bone marrow and the spleen [20], as well the number of cells of granulocytic and macrophage lineages in bone marrow cells growing in peritoneally implanted diffusion chambers [21]. A few years later, several studies evaluating hematological effects of β-glucan administration were carried out by Patchen’s group in Bethesda (MD, USA). In their initial studies using particulate glucan, stimulation of several progenitor cell lineages, namely of those for granulocytes, granulocytes/macrophages, macrophages, and erythrocytes, was observed [22,23,24]. Later findings of this research group based on experiments testing the hematopoiesis-modulating action of soluble β-glucan have shown nearly identical profile of stimulatory hematological effects of this β-glucan form on hematopoietic progenitor cells [25]. These results have been confirmed by Hashimoto *et al.* [26], who have also described an increased percentage of peripheral blood polymorphonuclear leukocytes in β-glucan-treated mice. The above-mentioned studies have opened the way to experiments aimed at testing β-glucan under conditions of myelosuppression of various etiologies.

## 3. Effects of β-Glucans on Radiation-Suppressed Hematopoiesis

Two research groups, namely those of Pospíšil and Hofer (Brno, Czech Republic), and that of Patchen, have performed most of the studies testing β-glucan’s effects on hematopoiesis damaged by ionizing radiation. Both groups have tested the actions of particulate as well as of soluble β-glucan.

Pospíšil *et al.* [27] have shown in 1982 that a single pre- or post-irradiation (^60^Co γ-rays) administration of particulate β-glucan enhances hematopoietic recovery in mice, as assessed by basic hematological paremeters like spleen weight, bone marrow cellularity, or peripheral blood granulocyte counts. Later it has been reported that soluble β-glucan stimulates hematopoiesis by increasing the bone marrow cellularity when given 24 hours before irradiation of mice with a nearly lethal dose of 7 Gy of γ-rays [28]. These data have been extended by findings of positive effects in mice given soluble β-glucan either in a single dose or repeatedly after their exposure to a sublethal radiation dose [29] (in this study also the compartments of bone marrow hematopoietic progenitor and precursor cells have been evaluated), or repeatedly after each of three to five fractional radiation doses [30]. Findings of the group of Pospíšil and Hofer group on hematopoiesis-stimulating effects of β-glucan have been summarized in a short review [31].

In 1982, *i.e.*, at the same time as Pospíšil *et al.* [27], the first report on hemopoiesis-enhancing effects of particulate β-glucan in radiation-suppressed hematopoiesis was reported by Patchen and MacVittie [32]; they have demonstrated that particulate β-glucan administered to mice after irradiation increases numbers of pluripotent hematopoietic stem cells in the spleen, as measured by the endogenous spleen colony technique. These authors also reported significant effects of particulate β-glucan on the state of mouse hematopoiesis evaluated by determining a wide spectrum of data on hematopoietic progenitor cells, when the drug was administered prior to sublethal or lethal radiation exposure [33,34,35,36]. In 1984, a study has been published on hematopoiesis-modulating action of nine different soluble polyglycans including several β-glucans administered before or after irradiation [37]; most of the β-glucans evaluated have shown hematopoiesis-stimulating effects at least comparable with those of particulate β-glucan. Soluble β-glucan has been also reported to amend hematopoiesis-suppressing effects of the quinolone antibiotic pefloxacin in irradiated mice [38].

Both research groups have also paid attention to the use of β-glucan in combined-treatment regimens. At least additive positive effects on hematopoiesis have been reported when pre-irradiation administrations of cystamine or WR-2721, the chemical radiprotectors, were combined with postirradiation treatment with particulate β-glucan [39,40]. A three-drug combination, namely that of particulate β-glucan, WR-2721 and selenium, has been shown to produce the highest numbers of hematopoietic progenitor cells and to induce the most extended survival of lethally irradiated mice in comparison with the effects of single compounds [41]. Soluble β-glucan has been reported to potentiate hematopoiesis-stimuating effects of granulocyte colony-stimulating factor (G-CSF) when both the substances were administered to mice in a post-irradiation exprimental setting [42]. Hematopoietic recovery in irradiated mice has been also significantly enhanced by a combination of soluble β-glucan and diclofenac, a cyclooxygenase inhibitor, in singly [43] and repeatedly irradiated mice [44].

The above-summarized convincing proofs of the positive effects of β-glucan on post-irradiation hematopoietic recovery obtained by the two laboratories have been also supported by other research groups. Thus, Líšková *et al.* [45] have published their findings on elevation of numbers of hematopoietic pluripotent stem cells (endogenous spleen colony-forming units) in sublethally irradiated mice by soluble β-glucan. A recent study by Cramer *et al.* [46] has also confirmed the stimulatory effects of soluble β-glucan on proliferation of hematopoietic progenitor cells and leukocyte recovery after sublethal irradiation of experimental mice.

## 4. Effects of β-Glucans on Hematopoiesis Suppressed by Cytotoxic Drugs

Findings from experiments aimed at modulation of cytotoxic drug-damaged hematopoiesis are important from a practical point of view because of the high number of patients in which bone marrow suppression represents an undesirable side effect of anti-cancer therapy. Two soluble β-glucans differing in the degree of substitution have been tested in 1993 for their ability to counteract cyclophosphamide (CY)-induced myelosuppression in mice and their effects on bone marrow and spleen cellularity, and on peripheral leukocyte numbers have been described as significantly positive, but modest [47]; in this study the administration of CY preceded that of β-glucan. When soluble β-glucan was administered to mice in a protective treatment regimen before CY, an expressive increase in bone marrow hematopoietic progenitor cells for granulocytes and macrophages (GM-CFC) has been found on days 4 and 8 after CY administration [48]. In CY-treated monkeys, one or two post-CY β-glucan intravenous infusions accelerated white blood cell recovery and reduced the median duration of neutropenia [48]. Numbers of GM-CFC have been also found to be significantly increased in doxorubicine-treated mice after administration of maitake (*Grifola frondosa*) β-glucan in comparison with saline-administered controls [49,50]. A recent study on mice conditioned by CY has revealed a stimulatory action of orally administered soluble β-glucan on monocyte production [51].

## 5. Other Hematological Effects of β-Glucans

Soluble β-glucan administered intravenously to mice has been reported to induce mouse hematopoietic stem cell proliferation and differentiation of progenitor cells for granulocytes and macrophages in umbilical cord blood [52] and to support homing and engraftment of hematopoietic stem cells [6]. The drug was also found to mobilize peripheral blood progenitor cells and to enhance G-CSF-mediated peripheral blood progenitor cell mobilization [53]. Oral administration of soluble β-glucan has been shown to positively modulate hematopoiesis in mice infected by Listeria monocytogenes, as assessed by evaluating numbers of GM-CFC [54].

## 6. Mechanisms of Hematopoiesis-Modulating Effects of β-Glucans

Mechanisms of the hematopoiesis-modulating actions of β-glucans have been investigated by both *in vitro* and *in vivo* experiments. A rather extensive amount of data exists on the ability of β-glucans to induce production of various cytokines and hematopoietic growth factors. It has been reported that both soluble and particulate β-glucans increase blood plasma concentration of interleukin-1 (IL-1) [55,56]. IL-1 seems to be of importance especially in connection with radioprotective effects of β-glucans on hematopoiesis (see above) and with the fact that this cytokine is known as a radioprotector of bone marrow [57]. Soluble β-glucan has been also found to stimulate directly production of G-CSF, an important hematopoietic growth factor, by monocytes [52]. Production of interleukin-6 (IL-6), another hematopoietic cytokine, has been positively influenced by soluble β-glucan in mice infected by *Listeria* monocytogenes in which the level of IL-6 was decreased by the infection [54].

Nevertheless, *in vitro* experiments of Turnbull *et al.* [58] have shown that β-glucan can enhance hematopoietic activity also independently of secondary cytokine stimulation. Cramer *et al.* [46] have emphasized the role of complement system in hematopoiesis-stimulating activities of soluble β-glucan: they report that β-glucan is a ligand of the complement receptor 3 lectin-like domain and that this substance enhances complement-mediated recovery after bone marrow injury. 

Patchen *et al.* [36] have stressed the free radical scavenging ability of β-glucan. This property of β-glucan may play an important role especially when administered before irradiation; it may thus act radioprotectively also as a chemical radioprotector.

## 7. Undesirable Side Effects of β-Glucans: Particulate *vs.* Soluble Glucan

As can be deduced from Section 2 to Section 4 of this review, the hematopoiesis-modulating effects of soluble and particulate β-glucans are comparable both qualitatively and quantitatively. However, important from the point of view of already realized or intended practical utilization of β-glucans in human clinical practice are their undesirable side effects which are connected with their immunohematomodulating properties.

Granulomas are foci of chronic inflammation characterized by accumulation and proliferation of leukocytes, mainly of the mononuclear type [59]. Administration of particulate β-glucan has been found to cause formation of hepatic [59] and pulmonary [60] granulomas. Production of granulomas by particulate β-glucan in mice has been observed to be strain-dependent and its intensity has been correlated with the intensity of the hematopoietic response [61]. Nevertheless, results of studies comparing the significance of this side effect between particulate and soluble β-glucans have led their authors to the conclusions that this phenomenon was either less pronounced [62] or was absent [63] in soluble β-glucan-treated mice. Subsequent publication paying attention to the topic of granuloma formation in the liver of soluble β-glucan-administered mice has revealed its transient occurrence [64]. Their number has been found to be significantly increased only in animals administered soluble β-glucan repeatedy (4x) in a high individual doses of 6 mg per mouse [29].

Administration of particulate β-glucan to mice infected by murine hepatitis virus has been reported to produce microcytic anemia and changes in ferrokinetics [65]. Repeated (5x) administration of soluble β-glucan to mice has been found to be associated with hyporesponsiveness of their myeloid progenitor cells in comparison with a single dose. This result suggests that the phenomenon of tolerance to repeated β-glucan injections may play a role here [66].

Williams *et al.* [67] reported in 1988 a detailed pre-clinical safety evaluation of soluble β-glucan. Their study was performed on mice, rats, guinea pigs, and rabbits. The authors concluded that “the systemic administration of soluble glucan, over a wide dose range, does not induce mortality or significant toxicity, an important consideration in preparing soluble glucan for parenteral administration to human populations”.

All the data on undesirable side effects of β-glucans, as presented in this chapter, show that its soluble forms should be used preferably in clinical practice and that soluble β-glucan is suitable for administration to humans.

## 8. β-Glucans in Humans

As follows from the preceding summarization, administration of soluble β-glucans to humans should be preferred, because they exhibit stimulatory effects on the immune and hematopoietic systems in a similar fashion in comparison with particulate β-glucan forms, but show much lower incidence and intensity of undesirable side effects. Already in 1990, Browder *et al.* [68] published positive findings about trauma patients treated with soluble β-glucan. The current indication spectrum for therapy with soluble β-glucans is wide, ranging from trauma and surgical patients [69,70] to cancer patients treated with cytotoxic anti-tumor drugs [5,71]. In some of these studies, positive effects of soluble β-glucan on hematopoiesis have been shown and acknowledged [5,71]. At present, especially neutral soluble yeast β-1,3;1,6-glucan preparations [e.g., 46,72] are considered for use in humans.

## 9. Conclusions

In the opinion of the authors of this review, the hematopoiesis-stimulating effects of soluble β-glucans together with their low toxicity predetermine these drugs to their ever increasing use in human medicinal practice. The authors are convinced that the effects of soluble β-glucans on radiation-suppresses hematopoiesis, as summarized in Section 3 of this review, should lead to considerations of incorporating them into the spectrum of drugs used for treating acute radiation disease, induced by high doses of ionizing radiation in emergencies caused by nuclear accidents or terrorist attacks. The data on effectiveness of β-glucans on hematopoiesis damaged by cytotoxic drugs, summed up in Section 4, also suggest their usefulness in counteracting the cytotoxic effects of chemotherapy used to treat patients with cancer.

## References

[1] Pilemer L., Ecker E.E. (1941). Anticomplementary factor in fresh yeast. J. Biol. Chem..

[2] Di Carlo F.J., Fiore J.V. (1958). On the composition of zymosan. Science.

[3] Riggi S.J., DiLuzio N.R. (1961). Identification of a reticuloendothelial stimulatory agent in zymosan. Am. J. Physiol..

[4] Dunlap J., Minami E., Bhagwat A.A., Keister D.L., Stacey G. (1996). Nodule development induced by mutants of Bradyrhizobium japonicum defective in cyclic B-glucan synthesis. Mol. Plant Microbe Interact..

[5] Magnani M., Castro-Gomez R.H., Aoki M.N., Gregorio E.P., Libos F., Watanabe M.A.E. (2010). Effects of carboxymethyl-glucan from Saccharomyces cerevisiae on the peripheral blood cells of patients with advanced prostate cancer. Exp. Ther. Med..

[6] Ohno N., Miura N.N., Nakajima M., Yadomae T. (2000). Antitumor 1,3-beta-glucan from cultured fruit body of Sparassis crispa. Biol. Pharm. Bull..

[7] Chang R. (2002). Bioactive polysaccharides from traditional Chinese medicine herbs as anticancer adjuvants. J. Altern. Complement. Med..

[8] Marklinder I., Johansson L., Haglund A., NagelHeld B., Seibel W. (1996). Effects of flour from different barley varieties on barley sour dough bread. Food Qual. Preference.

[9] Lin H., De Stanchina E., Zhou X.K., She Y., Hoang D., Cheung S.W.Y., Cassileth B., Cunningham-Rundles S. (2009). Maitake beta-glucan enhances umbilical cord blood stem cell transplantation in the DOD/SCID mouse. Exp. Biol. Med..

[10] DiLuzio N.R. (1983). Immunopharmacology of glucan: A broad spectrum enhancer of host defense mechanisms. Trends Pharmacol. Sci..

[11] DiLuzio N.R. (1985). Update on the immunomodulating activities of glucan. Springer Semin. Immunopathol..

[12] Kokoshis P.L., Williams D.L., Cook J.A., DiLuzio N.R. (1987). Increased resistance to Staphylococcus-aureus infection and enhancement in serum lysozyme activity by glucan. Science.

[13] DiLuzio N.R., Williams D.L. (1978). Protective effects of glucan against systemic Staphylococcus aureus septicemia in normal and leukemic mice. Infect. Immun..

[14] Williams D.L., DiLuzio N.R. (1980). Glucan-induced modification of murine viral-hepatitis. Science.

[15] Williams D.L., Cook J.A., Hoffmann E.O., DiLuzio N.R. (1978). Protective effects of glucan in experimentally induced candidiasis. J. Reticuloendothel. Soc..

[16] Browder I.W., Williams D.L., Kitahama A., DiLuzio N.R. (1984). Modification of postoperative C-albicans sepsis by glucan immunostimulation. Int. J. Immunopharmacol..

[17] Williams D.L., Mueller A., Browder W. (1996). Glucan-based macrophage stimulators: A review of their anti-infective potential. Clin. Immunother..

[18] Novelli E.M., Ramirez M., Civin C.I. (1998). Biology of CD34^+^CD38^−^ cells in lymphohematopoiesis. Leuk. Lymphoma.

[19] Grande T., Varas F., Bueren J.A. (2000). Residual damage of lymphohematopoietic repopulating cells after irradiation of mice at different stages of development. Exp. Hematol..

[20] Burgaleta C., Golde D.W. (1977). Effect of glucan on granulopoiesis and macrophage genesis in mice. Cancer Res..

[21] Niskanen E.O., Burgaleta C., Cline M.J., Golde D.W. (1978). Effect of glucan, a macrophage activator, on murine hemopoietic cell proliferation in diffusion chambers in mice. Cancer Res..

[22] Patchen M.L., Lotzová E. (1980). Modulation of murine hemopoiesis by glucan. Exp. Hematol..

[23] Patchen M.L., MacVittie T.J. (1983). Dose-dependent responses of murine pluripotent stem cells and myeloid and erythroid progenitor cells following administration of the immunomodulating agent glucan. Immunopharmacology.

[24] Patchen M.L., MacVittie T.J. (1983). Temporal response of murine pluripotent stem cells and myeloid and erythroid progenitor cells to low-dose glucan treatment. Acta Haematol..

[25] Patchen M.L., MacVittie T.J. (1986). Hemopoietic effects of intravenous soluble glucan administration. J. Immunopharmacol..

[26] Hashimoto K., Suzuki I., Ohsawa M., Oikawa S., Yadomae T. (1990). Enhancement of hematopoietic response of mice by intraperitoneal administration of a β-glucan, SSG, obtained from Sclerotinia sclerotiorum. J. Pharmacobio-Dyn..

[27] Pospíšil M., Jarý J., Netíková J., Marek M. (1982). Glucan-induced enhancement of hemopoietic recovery in gamma-irradiated mice. Experientia.

[28] Pospíšil M., Šandula J., Pipalová I., Hofer M., Viklická Š. (1991). Hemopoiesis stimulating and radioprotective effects of carboxymethylglucan. Physiol. Res..

[29] Hofer M., Pospíšil M., Viklická Š., Pipalová I., Holá J., Netíková J., Šandula J. (1995). Effects of postirradiation carboxymethylglucan administration in mice. Int. J. Immunopharmacol..

[30] Hofer M., Pospíšil M., Pipalová I., Holá J., Šandula J. (1995). Haemopoiesis-enhancing effects of repeatedly administered carboxymethylglucan in mice exposed to fractionated irradiation. Folia Biol. (Praha).

[31] Hofer M., Pospíšil M. (1997). Glucan as stimulator of hematopoiesis in normal and gamma-irradiated mice. A survey of the authors’ results. Int. J. Immunopharmacol..

[32] Patchen M.L., MacVittie T.J., Norman J.J., Sorkin E. (1982). Macrophages and Natural Killer Cells.

[33] Patchen M.L., MacVittie T.J. (1985). Stimulated hemopoiesis and enhanced survival following glucan treatment in sublethally and lethally irradiated mice. Int. J. Immunopharmacol..

[34] Patchen M.L., MacVittie T.J., Wathen L.M. (1984). Effects of pre- and post-irradiation glucan treatment on pluripotent stem cells, granulocyte, macrophage and erythroid progenitor cells, and hemopoietic stromal cells. Experientia.

[35] Patchen M.L., MacVittie T.J., Brook I. (1986). Glucan-induced hemopoietic and immune stimulation: Therapeutic effects in sublethally and lethally irradiated mice. Meth. Find. Exp. Clin. Pharmacol..

[36] Patchen M.L., D’Alesandro M.M., Brook I., Blakely W.F., MacVittie T.J. (1987). Glucan: Mechanisms involved in its "radioprotective" effect. J. Leukoc. Biol..

[37] Patchen M.L., DiLuzio N.R., Jacques P., MacVittie T.J. (1984). Soluble polyglycans enhance recovery from cobalt-60-induced hemopoietic injury. J. Biol. Response Mod..

[38] Patchen M.L., Brook I., Elliott T.B., Jackson W.E. (1993). Adverse effects of pefloxacin in irradiated C3H/HeN mice: Correction with glucan therapy. Antimicrob. Agents Chemother..

[39] Pospíšil M., Netíková J., Pipalová I., Jarý J. (1991). Combined radioprotection by preirradiation peroral cystamine and postirradiation glucan administration. Folia Biol. (Praha).

[40] Patchen M.L., D’Alesandro M.M., Chirigos M.A., Weiss J.F. (1988). Radioprotection by biological response modifiers alone and in combination with WR-2721. Pharmacol. Ther..

[41] Patchen M.L., MacVittie T.J., Weiss J.F. (1990). Combined modality radioprotection: The use of glucan and selenium with WR-2721. Int. J. Radiat. Oncol. Biol. Phys..

[42] Patchen M.L., MacVittie T.J., Solberg B.D., Souza L.M. (1990). Survival enhancement and hemopoietic regeneration following radiation exposure: Therapeutic approach using glucan and granulocyte colony-stimulating factor. Exp. Hematol..

[43] Pospíšil M., Hofer M., Pipalová I., Viklická Š., Netíková J., Šandula J. (1992). Enhancement of hematopoietic recovery in gamma-irradiated mice by the joint use of diclofenac, an inhibitor of prostaglandin production, and glucan, a macrophage activator. Exp. Hematol..

[44] Hofer M., Pospíšil M., Viklická Š., Vacek A., Pipalová I., Bartoníčková A. (1993). Hematopoietic recovery in repeatedly irradiated mice can be enhanced by a repeatedly administered combination of diclofenac and glucan. J. Leukoc. Biol..

[45] Líšková A., Wagnerová J., Červeňáková L., Krištofová A., Ferenčík M. (1990). The effect of soluble glucan derivates on spleen colony-forming units in sublethally irradiated mice. Folia Microbiol..

[46] Cramer D.E., Allendorf D.J., Baran J.T., Hansen R., Marroquin J., Li B., Ratajczak J., Ratajczak M.Z., Yan J. (2006). β-Glucan enhances complement-mediated hematopoietic recovery after bone marrow injury. Blood.

[47] Wagnerová J., Líšková A., Navarová J., Krištofová A., Trnovec T., Ferenčík M. (1993). The effects of two glucan carboxymethylderivatives with various substitution degress on cyclophosphamide immunosuppression in mice. Immunopharmacol. Immunotoxicol..

[48] Patchen M.L., Vaudrain T., Correira H., Martin T., Reese D. (1998). In vitro and *in vivo* hematopoietic activities of Betafectin^®^ PGG-glucan. Exp. Hematol..

[49] Lin H., She Y.-H., Cassileth B.R., Sirotnak F., Cunningham-Rundles S. (2004). Maitake beta-glucan MD-fraction enhances bone marrow colony formation and reduces doxorubicin toxicity *in vitro*. Int. Immunopharmacol..

[50] Ito K., Masuda Y., Yamasaki Y., Yokota Y., Nanba H. (2009). Maitake beta-glucan enhances granulopoiesis and mobilization of granulocytes by increasing G-CSF production and modulating CXCR4/SDF-1 expression. Int. Immunopharmacol..

[51] Harnack U., Eckert K., Fichtner I., Pecher G. (2010). Comparison of the effect of orally administered soluble beta-(1,3),(1,6)-D-glucan and of G-CSF on the recovery of murine hematopoiesis. In Vivo.

[52] Lin H., Cheung S.W.Y., Nesin M., Cassileth B.R., Cunningham-Rundles S. (2007). Enhancement of umbilical cord blood cell hematopoiesis by maitake beta-glucan is mediated by granulocyte colony-stimulating factor production. Clin. Vaccine Immunol..

[53] Patchen M.L., Liang J., Vaudrain T., Martin T., Melican D., Zhong S., Stewart M., Quesenberry P.J. (1998). Mobilization of peripheral blood progenitor cells by betafectin^®^ PGG-glucan alone and in combination with granulocyte colony-stimulating factor. Stem Cells.

[54] Torello C.O., de Souza Queiroz J., OLiveira S.C., Queiroz M.L.S. (2010). Immunohematopoietic modulation by oral β-1,3-glucan in mice infected with Listeria monocytogenes. Int. Immunopharmacol..

[55] Sherwood E.R., Williams D.L., McNamee R.B., Jones E.L., Browder I.W., Di Luzio N.R. (1987). Enhancement of interleukin-1 and interleukin-2 production by soluble glucan. Int. J. Immunopharmacol..

[56] Abel G., Czop J.K. (1992). Stimulation of human monocyte β-glucan receptors by glucan particles induces production of TNF-α and IL-1β. Int. J. Immunopharmacol..

[57] Dorie M.J., Allison A.C., Zaghloul M.S., Kallman R.F. (1989). Interleukin 1 protects against the lethal effects of irradiation of mice but has no effect on tumors in the same animals. Proc. Soc. Exp. Biol. Med..

[58] Turnbull J.L., Patchen M.L., Scadden D.T. (1999). The polysaccharide, PGG-glucan, enhances human myelopoiesis by direct action independent of and additive to early-acting cytokines. Acta Haematol..

[59] Deimann W., Fahimi H.D. (1980). Hepatic granulomas induced by glucan. An ultrastructural and peroxidase-cytochemical study. Lab. Invest..

[60] Kimura A., Sherwood R.L., Goldstein E. (1983). Glucan alteration of pulmonary antibacterial defense. J. Reticuloendothel. Soc..

[61] Pospíšil M., Tkadleček L., Netíková J., Pipalová I., Viklická Š., Kozubík A., Vácha J., Jarý J. (1988). Interstrain differences in the responsivenss of mice to glucan with respect to hematological effects and manifestations of late damage. Exp. Pathol..

[62] Bowers G.J., Patchen M.L., MacVittie T.J., Hirsch E.F., Fink M.P. (1986). A comparative evaluation of particulate and soluble glucan in an endotoxin model. Int. J. Immunopharmacol..

[63] Di Luzio N.R., Williams D.L., McNamee R.B., Edwards B.F., Kitahama A. (1979). Comparative tumor-inhibitory and anti-bacterial activity of soluble and particulate glucan. Int. J. Cancer.

[64] Baker W.H., Nold J.B., Patchen M.L., Jackson W.E. (1992). Histopathologic effects of soluble glucan and WR-2721, independently and combined in C3H/HeN mice. Proc. Soc. Exp. Biol. Med..

[65] Vácha J., Znojil V., Pospíšil M., Holá J., Pipalová I. (1994). Microcytic anemia and changes in ferrokinetics as late after-effects of glucan administration in murine hepatitis virus-infected C57BL/10ScSnPh mice. Int. J. Immunopharmacol..

[66] Pospíšil M., Vacek A., Hofer M., Viklická Š., Pipalová I., Šandula J.  (1993). Hyporesponsiveness of murine myeloid progenitor cells to glucan following its repeated administration. Folia Biol. (Praha).

[67] Williams D.L., Sherwood E.R., Browder I.W., McNamee R.B., Jones E.L., DiLuzio N.R. (1988). Pre-clinical safety evaluation of soluble glucan. Int. J. Immunopharmacol..

[68] Browder W., Williams D., Pretus H., Oliveiro G., Enrichens F., Mao P., Franchello A. (1990). Beneficial effect of enhanced macrophage function in the trauma patient. Ann. Surg..

[69] Spruijt N.E., Visser T., Leenen L.P.H. (2010). A systematic review of randomized controlled trials exploring the effect of immunomodulative interventions on infection, organ failure, and mortality in trauma patients. Crit. Care.

[70] Babineau T.J., Marcello P., Swails W., Kenler A., Bistrian B., Forse R.A. (1994). Randomized phase I/II trial of a macrophage-specific immunomodulator (PGG-glucan) in high-risk surgical patients. Ann. Surg..

[71] Weitberg A.B. (2008). A phase I/II trial of beta-(1,3)/(1,6) D-glucan in the treatment of patients with advanced malignancies receiving chemotherapy. J. Exp. Clin. Cancer Res..

[72] Hong F., Yan J., Baran J.T., Allendorf D.J., Hansen R.D., Ostroff R., Xing P.X., Cheung N.-K.V., Ross G.D. (2004). Mechanisms by which orally administered β-1,3-glucans enhance the tumoricidal activity of antitumor monoclonal antibodies in murine tumor models. J. Immunol..

